# A skellam model to identify differential patterns of gene expression induced by environmental signals

**DOI:** 10.1186/1471-2164-15-772

**Published:** 2014-09-08

**Authors:** Libo Jiang, Ke Mao, Rongling Wu

**Affiliations:** Center for Computational Biology, College of Biological Sciences and Technology, Beijing Forestry University, Beijing, 100083 China; Center for Statistical Genetics, The Pennsylvania State University, Hershey, PA 17033 USA

**Keywords:** RNA-seq, Skellam distribution, EM algorithm, *Arabidopsis thaliana* embryos

## Abstract

**Background:**

RNA-seq, based on deep-sequencing techniques, has been widely employed to precisely measure levels of transcripts and their isoforms expressed under different conditions. However, robust statistical tools used to analyze these complex datasets are lacking. By grouping genes with similar expression profiles across treatments, cluster analysis provides insight into gene functions and networks that have become increasingly important.

**Results:**

We proposed and verified a cluster algorithm based on a skellam model for grouping genes into distinct groups based on the pattern of gene expression in response to changing conditions or in different tissues. This algorithm capitalizes on the skellam distribution to capture the count property of RNA-seq data and clusters genes in different environments. A two-stage hierarchical expectation-maximization (EM) algorithm was implemented to estimate the optimal number of groups and mean expression levels of each group across two environments. A procedure was formulated to test whether and how a given group shows a plastic response to environmental changes. The model was used to analyze an RNA-seq dataset measured from reciprocal crosses of early *Arabidopsis thaliana* embryos that respond differently based on the extent of maternal and paternal genome contributions, from which genes associated with maternal and paternal contributions were identified. Simulation studies were also performed to validate the statistical behavior of the model.

**Conclusions:**

This model is a useful tool for clustering gene expression data by RNA-seq, thus facilitating our understanding of gene functions and networks.

## Background

The transcriptome is the total set of transcripts in a given organism at a specific developmental stage or under external environmental condition. Understanding the transcriptome is therefore essential to interpret the relationship between genome and organism function. Transcriptomics can be used to gain considerable biological insight by cataloguing all species of transcripts, determining the transcriptional structure of genes, and quantifying the changing expression levels of each transcript under various conditions [1–3]. RNA-seq, a next-generation sequencing technique, quantifies the transcriptome at a given moment in time, allowing for a better understanding of genome structure, gene expression patterns and gene regulatory networks [4, 5]. The organism can alter transcriptome levels and pattern responses to environmental changes [6, 7]. RNA-seq is a powerful tool used to identify specific genes associated with adaptive environments; such studies can assess genes involved in adaptation to environmental changes, particularly under different stresses or in various developmental stages. We hypothesized that, while an organism responds to growth conditions, particular environmental cues cause differential expression of its genes at a level that can be detected by RNA-seq. By profiling transcriptional changes induced by environmental changes, it is possible to identify gene regions or pathways that are likely to be targets of selection. This information is important to enable researchers to assess variation across gene regions, on a landscape scale, to predict the capacity of organisms to adapt to different conditions. Recently, RNA-seq experiments have evaluated differential mRNA processing events along the developmental gradient, as well as in different tissues, to account for the reaction norms of gene expression profiles [8–10]. In addition, RNA-seq has been used to assess the physiological response of organisms at different spatial scales and gain more insight into adaption mechanisms [11].

To better understand responses of gene expression to growth conditions, cluster analysis has been used as a powerful computational tool to divide genes into groups according to their expression patterns. In biology, cluster analysis is implied by the basic assumption that a gene expression profile may have similar features within the group [12–14]. Despite their widespread use, traditional approaches, such as hierarchical clustering algorithms and k-means algorithms, are largely heuristic, lacking a stringent inference about the underlying biological mechanisms. On the other hand, a model-based clustering approach assumes that the data are generated by a mixture of the underlying probability distribution components, in which a different group or cluster represents a component [15–18]. Also, this approach is flexible in choosing the component distribution and obtaining density estimation for each cluster. Nevertheless, most existing approaches for model-based cluster analysis have several limitations. First, the level of gene expression determined by RNA-seq is represented by the abundance of short reads, mapped to the reference, which is defined as a set of exons [19]. In practice, model-based cluster analysis is computationally difficult, especially because some genes are expressed at a very high level. In general, to discover important biological changes in expression and eliminate calculative hardship, normalization continues to be an essential step in the analysis, but most normalization methods neglect data features [20]. As a type of count data, three discrete probability distributions: binomial, Poisson and negative binomial (NB), have been used to model RNA-seq data [21–23].

Second, a regular RNA-seq experiment designed is to compare gene expression levels between test conditions. By comparing differential expression across treatments, one can characterize key genes that regulate the pattern of an organism’s response to rapid and stochastic environmental changes. Joint clustering for expression amounts in different treatments has been developed [24], but this strategy may not be sensitive to identify the differential response of genes to environmental changes, i.e., phenotypic plasticity [15]. The phenotypic plasticity of a gene can be expressed as the difference or ratio of expression amounts of the gene between two particular treatments. Since the difference and ratio of two Poisson variables requires totally different treatments of statistical modeling, we will, in this study, focus on model-based clustering for treatment-dependent differences to accommodate environmental impact.

Although some attempt has been made to overcome the first limitation [24], simultaneous treatment of the two limitations has not been explored in the literature. Here, we developed a computational model that clusters the differences between two statistically independent random variables, each having a Poisson distribution. Since the difference of two Poisson variables follows a skellam distribution [25], skellam parameters were implemented within a mixture model framework in which each component is represented by a distinct pattern of expression differentiation. Model parameters are estimated through the two-stage hierarchical expectation–maximization (EM) algorithm. Mean level of gene expression for a group is calculated for different environments, allowing us to compare the response level of gene expression to environmental changes. Results from this skellam model will obtain diverse insight into the genetic basis underlying adaptation to environments. The skellam model was used to analyze an RNA-seq dataset collected for early *Arabidopsis thaliana* embryos derived from reciprocal crosses in the one-to-two-cell stage [26]. By comparing it with conventional k-means and self-organizing mapping approaches, we show that the new model is statistically more powerful for gene clustering.

## Methods

### Mixture model-based likelihood

The most common type of transcriptome study is carried out to measure the response of organisms to two treatments. This type of analysis is especially useful for comparison of expression in different organs, treated versus untreated conditions in the same tissue, or studying the difference between reciprocal crosses, etc. Suppose we obtain a transcriptome dataset in which the organism is measured for reads of *n* genes with two treatments (1 and 2), and expression reads of gene *i* are denoted as *X*_*i*_ and *Y*_*i*_, respectively. In general, genes that are differentially expressed can be identified by determining differential expression between treatments. To assess gene expression changes across treatments, cluster analysis is a powerful tool for analyzing gene expression levels according to different patterns of gene expression. Therefore, we can discern different groups of genes per their functional similarities and differences in their plastic responses to changes in environment.

For any gene *i*, it should arise from one of the *J* groups that are classified on the basis of two expression values with two treatments. The joint likelihood of the expression data *z*_*i*_ = (*X*_*i*_ - *Y*_*i*_) of *n* genes is written as
1

where *θ* represents a set of unknown parameters, *π*_*j*_ represents the probability of group *j*(*j* = 1, …, *J*) in the total genes, and *f*_*j*_(*z*_*i*_) represents the density function of two expression difference values for gene *i* that belongs to group *j* with the two treatments.

We used a skellam distribution function to describe *f*_*j*_(*z*_*i*_), which is specified by the mean values of gene expression with treatment 1(*θ*_*j*1_) and 2(*θ*_*j*2_). Let *X*_*i*_ and *Y*_*i*_ denote two independent random Poisson variables with mean *θ*_*j*1_, *θ*_*j*2_ for group *j*, respectively. The two variables are expressed as one independent random variable: z_*i*_ = *X*_*i*_ - *Y*_*i*_. A skellam distribution of *z*_*i*_ for gene *i* is described by a probability density function, expressed as
2

where *θ*_*j*1_ and *θ*_*j*2_ represent the mean expression values of all genes that belong to group *j* in treatment 1 and 2, respectively, with the two parameters arrayed in Λ_*J*_ = (*θ*_*j*1_, *θ*_*j*2_). Here, *f*_*j*_(*z*_*i*_) in the mixture model (1) is specified by *f*_*j*_(*Z* = *z*_*i*_|*Λ*_*j*_).

### Estimation via the EM algorithm

Maximum-likelihood (ML) estimation is more complicated since the likelihood involves the modified Bessel function. If the true data X_*i*_ and Y_*i*_ are observed, then the estimation is straightforward since their means would be the ML estimates for Poisson parameters. Here, an EM-type algorithm is constructed based on the missing data representation of difference values *Z*. Unlike a general skellam model, the likelihood of *z*_*i*_ is formulated within a mixture-model framework (1), whose estimation is based on implementation of the EM algorithm. Thus, we implemented a two-stage hierarchical EM algorithm to estimate the parameters Λ_*j*_ of the likelihood (1).

In the E step, we calculate the conditional expectation of *X*_*i*_ by
3

where f* is the density of joint distribution of (*X*_*i*_, *Y*_*i*_). Meanwhile, we calculate the posterior probability of gene *i* that belongs to group *j*,
4

In the M step, we obtained the estimates of parameters *π*_*j*_ and Λ_*j*_ by using
567

where E and M steps are iterated between equations (3–7) until the estimates of the unknown parameters converge to stable values. Estimates obtained this way represent the maximum-likelihood estimates (MLEs) of the parameters.

### Choosing an optimal number of groups

One important question in the implementation of model-based clustering analysis is to determine the actual number of clusters using a model selection criterion, such as BIC. For a given number of clusters *J*, we calculate the likelihood *L* by (1) and the BIC by - 2 log(*L*) + *J* log(*n*), where *n* is the number of genes in the model. A low value of BIC corresponds to an optimal number of clusters.

### Hypothesis tests

After an optimal number of gene clusters is determined, we tested whether genes are expressed differentially between treatments. Three biologically meaningful tests were formulated as follows:
8

(i) For a given group *j*, we want to know whether its genes are differently expressed between the two treatments. This can be tested using the following equation:

If the *H*_0_ is accepted, then the group of genes expressed between the two treatments is stable. Otherwise, they exhibit differential expression across treatments, in which case, they can be used as a predictor of environmental-induced changes.
9

(ii) For a pair of groups *j* and *l*, we want to know whether they interact with each other to determine environmental-induced changes. This can be determined using the following equation:

If the *H*_0_ is rejected, then these two groups of genes have significant interaction effects on biological changes between treatments.
10

(iii) For a particular group *j*, we want to know whether changes in gene expression for a group are consistent with the extent of change of the environment. This can be determined using the following equation:

where *c* represents the difference between the environmental signals between treatments. If *H*_0_ is rejected, then the change in gene expression for the group is consistent with a change in the environment between treatments.

For each of the hypotheses (8–10), the likelihood ratio test statistics (LR) between these two hypotheses *H*_0_ and *H*_1_ are calculated. Since the *H*_0_ is nested within *H*_1_, the LR value can be thought of being chi-square distributed, with the degree of freedom equaling the difference between the numbers of parameters to be estimated under the two hypotheses. The LR value is compared with a critical threshold to determine the acceptance or rejection of the null hypothesis. If these tests are incorporated by a particular environmental signal, e.g., temperature or nutritional level, we can better understand the relationship between gene expression and environmental change.

## Results

### Working example

The prevailing theory for the maternal-to-zygotic transition in plants proposes that most early embryonic mRNAs are maternally derived, resulting either from maternal inheritance or from higher transcriptional activity of maternally derived genes until the globular stages. However, this theory is difficult to reconcile with reports of equivalent maternal and paternal expression of interrogated genes at the preglobular stage. Recently, a study aimed to determine the origins of embryonic transcripts globally by reciprocally crossing polymorphic Col-0 and Cvi-0 *Arabidopsis thaliana* accessions Col-0 × Cvi-0 and Cvi-0 × Col-0; the transcriptomes of embryos with one-to-two cells were then measured for two reciprocal crosses [26], from which a total of 1,521 differential expression genes were gained.

**Figure 1:**
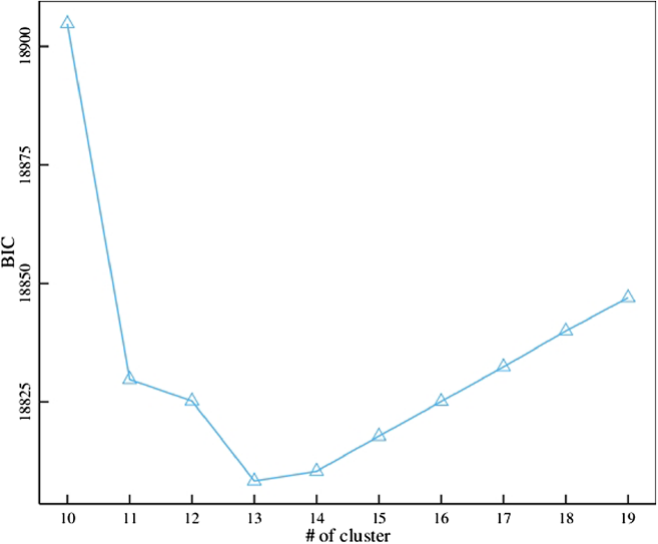
**Plot of BIC values over the number of groups calculated from the transcriptomic data of early** ***Arabidopsis thaliana*** **embryos in reciprocal crosses.**

**Figure 2:**
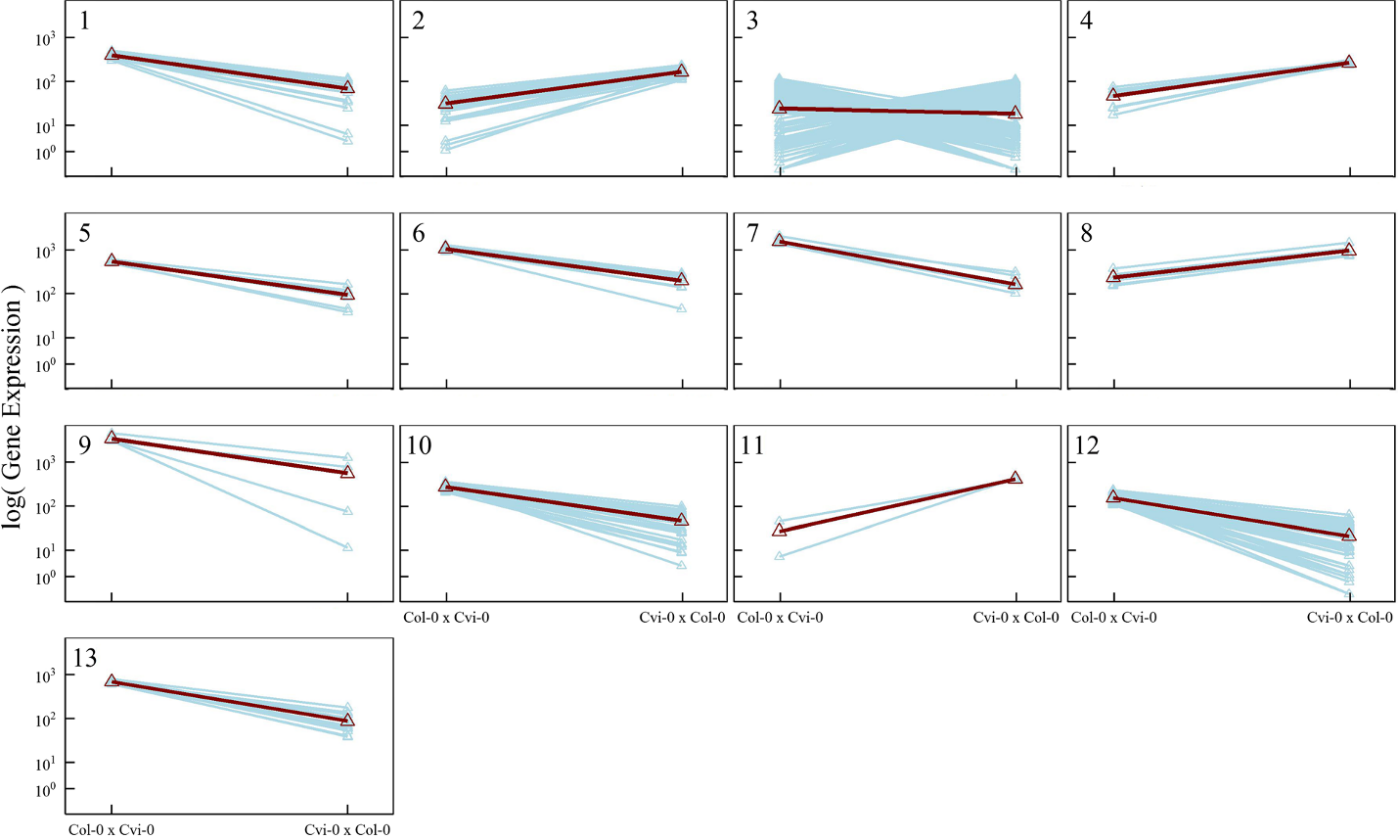
**Differentiation patterns of genes from 13 distinct groups expressed in early** ***Arabidopsis thaliana*** **embryos of reciprocal crosses.** In each group, the mean expression curve is indicated by a thick line over expression curves of individual genes (thin lines).

**Figure 3:**
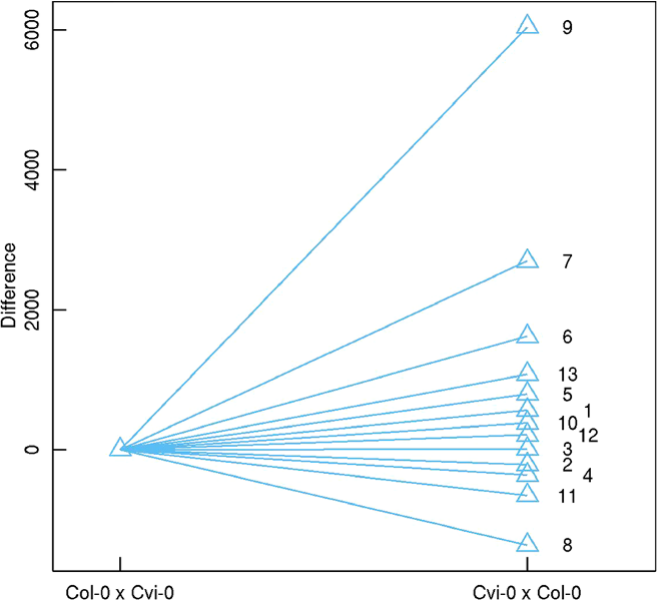
**Relative differences among gene expression curves of different groups expressed in early** ***Arabidopsis thaliana*** **embryos of reciprocal crosses.**

The skellam model was used to analyze this data, clustering 1521 DE genes into distinct groups. We used BIC to determine an optimal number of gene groups. From the plot of BIC value against the number of groups, 13 was found to be an optimal number of groups (Figure 1). For each group *j*, the mean values of gene expression (*θ*_*j*1_ and *θ*_*j*2_) in reciprocal crosses were estimated, with reasonable good standard errors, by a resampling approach (Table 1). In practical calculations, the estimate of *θ*_*j*_ is sensitive to the choice of initial values. To obtain a global maximum, multiple initial values have been selected and compared. Figure 2 illustrates mean expression values of each group in two crosses; 13 groups not only display differential levels of gene expression, but also vary dramatically in terms of the difference of expression between reciprocal crosses. In Figure 3, we showed the pattern of how genes are differently expressed over different crosses. As can be seen, 13 groups of genes did not parallel, exhibiting significant gene–environment interactions under reciprocal cross conditions.

**Table 1:** **Maximum likelihood estimates of mean expression values of genes (** ***θ***_***j*1**_ **and** ***θ***_***j*2**_ **,** ***j*** **= 1, …, 13) for 13 distinct groups in reciprocal crosses of early** ***Arabidopsis thaliana*** **embryos**

Group	Proportion	*θ*_*j*1_	*θ*_*j*2_
1	0.01287(0.0021)	2079.323(127.271)	1531.356(123.073)
2	0.02995(0.0034)	1679.736(119.864)	1869.816(129.490)
3	0.84635(0.0020)	1775.796(124.636)	1767.683(124.244)
4	0.00724(0.0007)	1615.983(122.105)	1947.895(127.480)
5	0.00445(0.0008)	2259.002(137.770)	1477.989(134.481)
6	0.00658(0.0008)	5565.413(1236.57)	3943.197(1221.47)
7	0.00460(0.0008)	15070.72(4277.45)	12378.64(4240.52)
8	0.00329(0.0009)	12640.44(4880.39)	14001.97(4875.96)
9	0.00263(0.0004)	63549.43(22325.8)	57509.99(22313.7)
10	0.02102(0.0020)	1977.736(130.340)	1597.144(120.552)
11	0.00132(0.0006)	2368.24(2138.77)	3025.391(2244.53)
12	0.05259(0.0045)	1874.926(129.278)	1676.266(120.332)
13	0.00721(0.0009)	3017.567(266.219)	1938.277(260.411)

The MSEs (in parentheses) of the estimates are calculated from 1000 bootstrapping samples.

The hypothesis test (8) provided information regarding the significance of expression differences between treatments to determine the extent of the maternal and paternal contributions. Of these 13 groups, gene expression levels from group 3 (accounting for nearly 84% of genes) tended to be stable between reciprocal crosses, although change in gene expression was statistically significant (*P* < 0.05) (Table 2). This indicates that most genes of maternal and paternal genomes contribute slightly differently to *Arabidopsis thaliana* embryos at the one-to-two cell stage. Approximately 6% of genes (groups 1, 5, 6, 7, 9, 10, 12, and 13) and about 10% (groups 2, 4, 8, and 11) were clearly down- or up-regulated from Col-0 × Cvi-0 to Cvi-0 × Col-0, respectively, suggesting that they were preferentially inherited from one parent in one-to-two cell embryos. Hypothesis test (9) was used to determine whether a particular pair of gene groups interacts with the environment. Table 3 lists the significance test used for such gene–gene interactions. All pairs of gene groups exhibited significant gene–environment interactions (*P* < 0.05). Hypothesis test (10) was utilized to investigate whether gene expression was consistent with environmental change. Except for group 2, all groups conform to the extent of environmental change (Table 4). All calculations and hypothesis tests done above took about 24 h in a 225-nodes computing cluster.

**Table 2:** **Hypothesis tests for gene–environment interactions between the two treatments in a group**

Group	Test static	*P*-value	FDR
1	1446.04	0.00	0.00
2	496.42	0.00	0.00
3	24.23	8.53e-07	8.53e-07
4	271.19	0.00	0.00
5	836.91	0.00	0.00
6	2358.29	0.00	0.00
7	1678.18	0.00	0.00
8	329.24	0.00	0.00
9	1146.73	0.00	0.00
10	1260.75	0.00	0.00
11	142.87	0.00	0.00
12	827.88	0.00	0.00
13	2115.68	0.00	0.00

**Table 3:** **Hypothesis tests for gene–environment interactions for different pairs of gene groups**

Group	Test static	P-value	FDR
1 versus 2	229.67	0.00	0.00
2 versus 3	13818.97	0.00	0.00
3 versus 4	14171.06	0.00	0.00
4 versus 5	24.06	9.32e-07	1.12e-06
5 versus 6	78.20	0.00	0.00
6 versus 7	4.13	4.22e-02	4.22e-02
7 versus 8	41.70	1.07e-10	1.43e-10
8 versus 9	15.08	1.03e-04	1.12e-04
9 versus 10	287.59	0.00	0.00
10 versus 11	344.34	0.00	0.00
11 versus 12	704.90	0.00	0.00
12 versus 13	595.10	0.00	0.00

**Table 4:** **Hypothesis test about whether gene expression is consistent with the change of environment**

Group	Test static	P-value	FDR
1	1.17	0.28	0.91
2	5.90	0.015	0.19
3	0.13	0.72	0.99
4	2.22	0.14	0.61
5	0.33	0.57	0.99
6	0.089	0.77	0.99
7	0.26	0.61	0.99
8	0.17	0.68	0.99
9	0.023	0.88	0.99
10	0.094	0.76	0.99
11	0	1.00	1.00
12	3.63	0.056	0.36
13	0.0096	0.92	0.99

**Figure 4:**
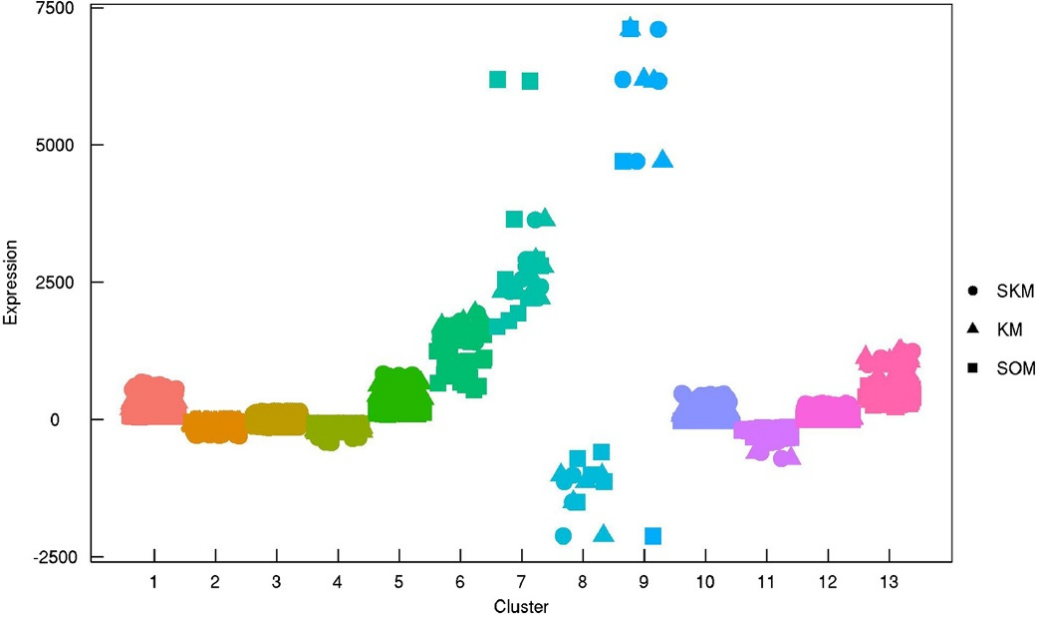
**Comparison of clustering results in 13 distinct groups using three methods (SKM: skellam model, KM: k-means, SOM: self-organizing map).**

The data were also analyzed by traditional approaches, k-means and self-organization mapping (SOM). K-means is a partitioning approach, whereas SOM is a method based on a machine learning algorithm that uses a competition and cooperation mechanism to achieve unsupervised learning, processed as implemented in the R package yasomi [27, 28]. It was observed that k-means and the skellam model produce a similar result, different from that by SOM (Figure 4). Since these three approaches have different underlying principles, they can be interpreted differently. K-means clustering tends to identify clusters of similar spatial extents, whereas SOM is typically used as an artificial neural network that is trained using unsupervised learning to produce a low-dimensional, discretized representation of the input space of the training samples. The skellam model identifies clusters based on their pattern of gene expression in response to treatment.

### Computer simulation

Simulation studies were conducted to test the statistical power of the skellam model. By assuming three up- or down-regulated expression patterns, we simulated 2000 genes expressed in two treatments. The treatment-dependent means of groups and their probabilities were given in Table 5.

**Table 5:** **Cluster parameter of the simulation study**

Group *j*	Treatment		*π*_*j*_
	*θ*_*j*1_	*θ*_*j*2_	
1	30	25	0.2
2	15	45	0.5
3	60	8	0.3

**Figure 5:**
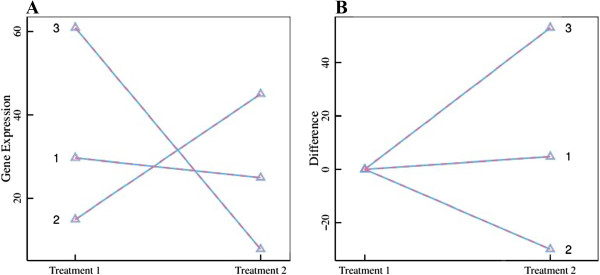
**Comparison of estimated gene expression curves (solid lines) with true curves (broken lines) for three distinct groups from the simulated data. (A)** Absolute values of gene expression in two treatments. **(B)** Relative differences between gene expression levels of two treatments.

Table 6 gives the maximum-likelihood estimates of *θ*_*j*1_ and *θ*_*j*2_, in a comparison with their true values. In general, mean gene expression values in different treatments can be reasonably well estimated. The estimated curves of gene expression for each group were broadly consistent with the true curves (Figure 5), suggesting that our model was fully powered.

**Table 6:** **Results of parameter estimates from simulated data**

Group	Proportion		*θ*_*j*1_		*θ*_*j*2_	
	True	MLE	True	MLE	True	MLE
1	0.2	0.201(0.004)	30	29.7(0.497)	25	25.0(0.501)
2	0.5	0.500(0.004)	15	16.1(0.439)	45	46.1(0.431)
3	0.3	0.299(0.004)	60	61.7(0.698)	8	8.59(0.693)

The MLE from the model are compared with the true values for each parameter. MSEs of the MLEs (in parentheses) are calculated from 1000 simulation replicates.

We used k-means and SOM to analyze the same simulation data. Overall, the skellam model performs better than SOM since the former correctly clusters all genes into their underlying groups whereas the latter provides incorrect clusters for about 20% of genes. Like the skellam model, K-means can correctly discern three groups and clusters all genes into correct groups. The advantage of skellam over k-means lies in its capacity to provide biologically testable hypotheses (8) – (10), thus being of greater value from a biological perspective.

## Discussion

Recently, RNA-seq has become a highly popular technology for measurement of transcript levels in response to different environment conditions. Here, we propose a statistical model to group RNA-seq data in response to changing environmental conditions based on a skellam distribution. The skellam model is able to identify and cluster co-expression patterns of genes derived from different treatments. The same group of co-regulated genes responds to environmental change through a similar function; therefore, a set of model responses can be estimated and tested in a functional space. These can then be used to characterize the functional relationship between genes and the environment. The model has three features that differentiate it from traditional clustering methods. First, traditional methods cluster genes based on their expression at single points in time or their joint expression at multiple points in time [22], ignoring the mechanism by which genes are differentially expressed in response to environmental conditions. By determining the differences in expression among treatments as the expression plasticity of a gene, the new model clusters genes into different groups based on their capacity to respond to environmental changes. This peculiarity makes the model particularly useful for understanding the changes in gene expression in response to different treatment conditions.

Second, classical clustering approaches are based largely on continuous expression data measured by microarrays [29, 30], whereas gene reads measured by RNA-seq are count data, which are believed to follow a Poisson distribution [20]. Our model has considered the Poisson property of reads. Third, the skellam model treats the co-expression of genes under different condition as a system and integrates their capacity to co-respond to environmental changes into clustering procedures. This treatment facilities our understanding of gene plasticity induced by environmental cues.

The skellam model has successfully clustered genes of early *Arabidopsis thaliana* embryos into groups based on their response to different conditions. Of the genes with a statistically significant change, group 9 is associated with adenosine triphosphate (ATP)-involved ATP synthase 9, ATP synthase subunit C family protein and ATPase, F1 complex, alpha subunit protein [31], and group 8 is related to arabinogalactan protein 21, pathogenesis-related thaumatin-like protein, and ribonuclease 1 [32]. Although both maternal and paternal genomes are active and contribute substantially to the embryonic transcriptome during the one-to-two-cell stage, some active gene sets are clearly derived from one parent.

We provided a general framework for gene clustering based on the Poisson function. Given a complex data with great variability in different treatments, i.e., overdispersion, the Poisson distribution with one free parameter is too simple to allow for the variance to be adjusted independently of the mean for such a data. Other more sophisticated distributions should be incorporated to provide a better flexibility of fit. These include negative binomial distribution as a natural extension of Poisson distribution and generalized Poisson distribution [33]. In general, clustering of genes with differential expression is not the final step of the analysis. Other analyses, such as gene set testing, gene network construction and knowledge databases should follow. A comprehensive model of integrating gene clustering and these follow-up analyses should be derived, which would enable geneticists to extract biological insight from gene expression data.

We used the difference of gene expression as a measure of gene plasticity over different environments. This measure can characterize the amount of environment-induced response, but it cannot well discern the slope of differentiation expression, i.e., the sensitivity of a gene environmental change per its expression unit). Such a slope can be described by the ratio of gene expression over different environments. In theory, the clustering model can be extended to cluster genes expressed under multiple conditions, and provides greater understanding of the mechanistic relationships between gene expression and environmental changes. The extended model allows for the classification of different trajectories of reaction norm in response to an environmental gradient. In addition, most studies of gene expression by RNA-seq are performed in a static state, but the role of dynamic gene expression in constructing regulatory networks is being recognized [14, 15]. To model dynamic changes in gene expression in response to environmental stimuli, more advanced statistical model such as longitudinal data analysis integrating the multivariate skellam distribution [34] is required; this warrants further investigation.

## Conclusion

As a deep-sequencing technique, RNA-seq has proven to be powerful for precisely measuring levels of transcripts and their isoforms expressed under different conditions. We have developed a computational algorithm that clusters genes into distinct groups based on the differences of RNA counts between different treatments. The algorithm is based on the Poisson distribution of counts, making use of the skellam function that specifies the distribution of the differences between two independent Poisson variables. A two-stage hierarchical EM algorithm was implemented to estimate the optimal number of groups and mean expression levels of each group across two environments. In a comparison with traditional clustering approaches, such as k-means and self-organization mapping, the new skellam model has more biological relevance, equipped with a capacity to test whether a given group is responsive to environmental changes and how this plastic response is related with, or induced by, an environmental cue. The skellam model provides a useful tool for clustering gene expression data by RNA-seq, thereby enhancing our understanding of gene functions and networks.
